# Reversible Growth of Gold Nanoparticles in the Low-Temperature Water–Gas Shift Reaction

**DOI:** 10.1021/acsnano.2c06504

**Published:** 2022-08-25

**Authors:** James H. Carter, Ali M. Abdel-Mageed, Dan Zhou, David J. Morgan, Xi Liu, Joachim Bansmann, Shilong Chen, R. Jürgen Behm, Graham J. Hutchings

**Affiliations:** † Max Planck-Cardiff Centre on the Fundamentals of Heterogeneous Catalysis FUNCAT, Cardiff Catalysis Institute, School of Chemistry, Cardiff University, Cardiff CF10 3AT, United Kingdom; ‡ Institute of Surface Chemistry and Catalysis, Ulm University, Albert-Einstein-Allee 47, D-89081, Ulm, Germany; § Leibniz Institute for Catalysis (LIKAT Rostock), D-18059 Rostock, Germany; ∥ DENSsolutions B.V., Delft 2628 ZD The Netherlands; ⊥ School of Chemistry and Chemical Engineering, In situ Center for Physical Sciences, Shanghai Jiao Tong University, 800 Dongchuan Road, Minhang District, Shanghai, China, 200240

**Keywords:** Gold, water−gas shift, X-ray absorption, *in situ*, electron microscopy, spectroscopy

## Abstract

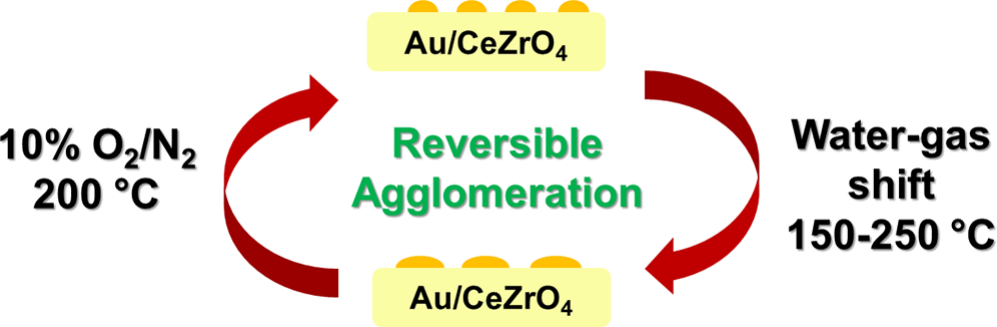

Supported gold nanoparticles are widely studied catalysts and are among the most active known for the low-temperature water–gas shift reaction, which is essential in fuel and energy applications, but their practical application has been limited by their poor thermal stability. The catalysts deactivate on-stream via the growth of small Au nanoparticles. Using operando X-ray absorption and in situ scanning transmission electron microscopy, we report direct evidence that this process can be reversed by carrying out a facile oxidative treatment, which redisperses the gold nanoparticles and restores catalytic activity. The use of *in situ* methods reveals the complex dynamics of supported gold nanoparticles under reaction conditions and demonstrates that gold catalysts can be easily regenerated, expanding their scope for practical application.

Supported nanoparticle catalysts play a crucial role in large scale chemical processes and underpin many modern manufacturing techniques. In particular, Au has emerged as a highly active metal for catalysis and has been commercialized in the hydrochlorination of acetylene.^1^ Nanoparticles (NPs) of Au are also known for their high activity in room-temperature CO oxidation^2^ and in the low-temperature water–gas shift (LT-WGS) reaction.^3^ The latter reaction is a key step in the production and ultrapurification of H_2_ feed gases used in hydrogen fuel cells and for the production of ammonia.^4^ Au dispersed on reducible metal oxide supports are among the most active LT-WGS catalysts so far known.^5^ However, supported Au catalysts, in particular on Ce-based materials, tend to deactivate rapidly on-stream, which limits their practical application. The deactivation of supported gold catalysts has been investigated and various mechanisms proposed. Evidence for the growth of Au species under reactions conditions has been shown, as well as the blocking of active sites by surface species such as carbonates.^6,7^ Attempts to reactivate supported Au catalysts have also been reported. Flytzani-Stephanopoulos and co-workers reported that O_2_ treatment of Au/CeO_2_ at 400 °C could restore the catalytic activity, and it was shown that this reoxidized both the support and a significant fraction of the Au. The authors suggested that the O_2_ treatment redispersed the Au based on temperature-programmed reduction, but no direct evidence was found. Karpenko showed that H_2_O was more effective than O_2_ in restoring activity,^8^ while Goguet et al. reported that increased H_2_O concentration in the reaction feed accelerated catalyst deactivation.^7^ Overall there is no consensus on the fundamental dynamics of Au NPs under different environments and temperatures. This has largely been due to the difficulty in measuring such changes and the need for *in situ* or *operando* measurements to directly probe the catalysts under working conditions.

One of the frontiers in heterogeneous catalysis is understanding the structural dynamics of NPs under reaction conditions. As characterization technology has improved, the understanding of the structure and morphology of active Au species during catalytic reactions has greatly advanced. Where it was originally thought that Au NPs several nanometers in diameter were the active species, aberration-corrected electron microscopes that afford atomic resolution have revealed the presence of subnanometer clusters, rafts, oligomers and atomically dispersed Au species, which can play a significant or even decisive role in the activity of these catalysts.^9,10^ The advent of the closed cell *in situ* scanning transmission electron microscopy (*in situ* STEM) with gas environments has elevated our knowledge of the morphology and dynamics of nanoparticle catalysts. *In situ* STEM enables a sample to be imaged while simultaneously exposed to more realistic thermal and gas environments, which is essential for gaining an accurate understanding of catalyst structure during chemical reactions. Furthermore, *in situ* and *operando* spectroscopy has equally advanced our understanding of the active structure of catalysts under reaction conditions. Specifically, *operando* X-ray absorption spectroscopy enables the oxidation state, co-ordination number and local environment of elements to be observed during the reaction.

In this work, we combine bulk sensitive *operando* XAS and *in situ* STEM with complementary surface sensitive X-ray photoelectron spectroscopy (XPS) measurements to study the activation, deactivation and regeneration of Au/CeZrO_4_ catalysts during the LT-WGS reaction. These experiments are supported with fast-reaction kinetic measurements during the initial activation phase of the LT-WGS reaction (1–10 min on stream). We demonstrate that under present reaction conditions, Au NPs grow and the catalyst deactivates, but an oxidative heat treatment at moderate temperature can redisperse a significant fraction of the highly active small Au NPs (<2 nm), which recovers the catalyst activity.

## Results and Discussion

**Figure 1:**
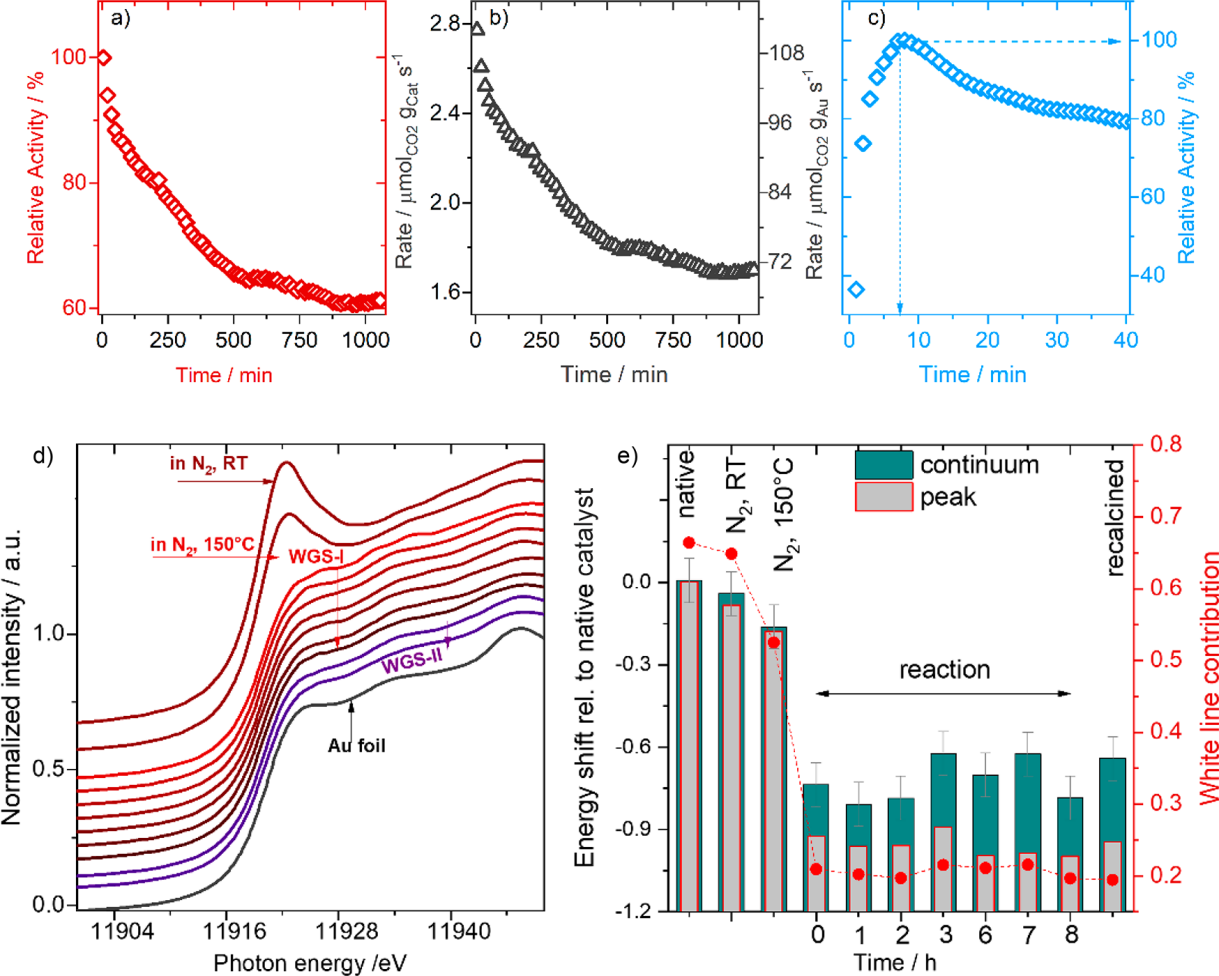
Catalytic activity and operando XAS during LT-WGS (a) Relative WGS reaction rate with time (rate normalized by initial rate) on Au/CeZrO_4_ catalyst at 150 °C in reformate gas (2% CO, 8.1% H_2_, 7.5% H_2_O and balance N_2_ – 50 N ml min^–1^) using gas chromatography (Δ*t* = 15 min). (b) Catalyst and gold mass normalized LT-WGS reaction rate at 150 °C. (c) Relative WGS activity measured using IR spectrometry with higher time resolution (Δ*t* = 1 min). (d) XANES spectra on the fresh Au/CeZrO_4_ catalyst at 28 and 150 °C in N_2_ (brown) and during subsequent reaction (WGS-I) and after a recalcination step (WGS-II) (45 min in 10% O_2_/N_2_ at 200 °C). (e) Energy shifts (columns) derived from a linear combination fit (assuming a Gaussian peak for the white line and an arctan function for transitions into continuum states) with respect to those recorded for the fresh Au/CeZrO_4_ sample. The negative sign denotes shifts of both contributions (peak and continuum states) to lower photon energies.

Kinetic measurements were first performed in a flow microreactor after drying 2.5 wt% Au/CeZrO_4_ catalyst at 150 °C under a flow of N_2_ for 30 min, using gas chromatography measurements for the calculation of reaction rates. The results showed a continuous deactivation for over 800 min on stream (see Figure 1a) with the relative activity decaying from 100% to about 60% over this period of time, after which no measurable changes could be observed. Changes in the catalyst and Au-mass normalized activity are represented in Figure 1b. These observations are in good agreement with previous findings on a similar Au/CeZrO_4_ catalyst where the catalyst showed quantitatively similar deactivation rate over the same period of time.^6,7^ These results are also in agreement with behavior observed on an Au/CeO_2_ catalyst under idealized conditions at 180 °C.^10,11^

We performed similar measurements during the XAS studies (described below) using IR spectroscopy for analysis with the advantage of a higher time resolution (Δt, 1 min), which is applied to accurately assign the time needed to reach the highest WGS activity. This allowed us to monitor the changes in the reaction behavior in the initial reaction phase, which was not possible in earlier studies. As shown in Figure 1c, there is a rather quick activation phase in the initial 12 min of the reaction. Activity measured after 1 min indicated the increase of relative activity from 35 (equiv. to 34.6% CO conversion, see Figure S1) to 100% after 7 min (equiv. to 98.8% CO conversion). At extended reaction time, a similar behavior was also reproduced on the same catalyst (see Figure S1). Obviously, there is a quick structural/electronic change of Au species taking place in the initial short reaction phase (1–7 min), which is followed by the continuous deactivation. Also, the deactivation from 7 to 25 min is much steeper than in the rest of the reaction period, which was previously ascribed to rapid agglomeration of small Au NPs during this initial period.^6^

Our recent investigations into the deactivation of Au/CeZrO_4_ during the WGS reaction used stop-start HAADF STEM to follow the agglomeration of Au NPs during the reaction. However, this approach required the removal of the catalyst from the reaction environment and inevitably samples only a small fraction of the whole sample. Therefore, we employed *operando* XAS to measure the change in the catalyst structure under working conditions. Such an approach has been successfully applied to monitor changing Au species over Au/CeO_2_ catalysts during CO oxidation and regeneration.^12^

Initially, we used *operando* XANES to follow the changes at the Au L_III_ edge to identify changes in the chemical/electronic state of the Au during the reaction. XANES spectra collected on the fresh catalyst after purging with a flow of N_2_ for 30 N ml min^–1^ at room temperature (28 °C) indicated the presence of Au species in Au/CeZrO_4_ catalyst in an almost completely oxidized state (i.e., as Au^3+^ ions). Heating up to 150 °C under continuous flow of N_2_ did not result in any significant change in the state of the catalyst (see Figure 1d). With the onset of the WGS reaction the first XANES spectrum recorded during the reaction (2–7 min of WGS reaction) indicated an abrupt and very quick disappearance of the white line, characteristic of the presence of Au^3+^ /Au^δ+^ ions. Both observations are in close agreement with previous findings for Au/CeO_2_ catalysts.^11^

Analysis of the CO conversion during these measurements for over 600 min on-stream showed similar behavior to that detected in the microreactor, except for a slightly faster decay of the activity in the first 100 min, probably due to the much higher CO conversion encountered in the XAS reaction cell (see Figure S1), and possibly due to the different geometry of the reactor. At extended reaction time (up to 11 h on-stream), the XANES spectra did not change much, indicating the stability of the oxidation state of Au during reaction (see Figure 1d).

For a quantitative interpretation of these changes, we derived changes in the position of the white line in the collected spectra (fresh catalyst and those during WGS reaction) with respect to the fresh sample (fully oxidized sample) (Figure 1e). Relative to the fresh sample, the energy shifted by about −0.2 eV for the sample heated in N_2_ at 150 °C, which increased to about −0.8 eV during the WGS reaction and remained stable until the end of the reaction (−0.8 ± 0.1 eV), hinting to a significantly more reduced state during reaction than in the fresh catalyst or after N_2_-treatment. Similarly, the contribution of the white line with its Gaussian shape at about 11921 eV to the reaction spectra (with respect to the fresh sample) decreased in a comparable manner, also indicating a strong reduction of the catalyst during reaction (see Figure 1d). Specifically, we see that the contribution of the Gaussian peak is even less than that of the spectra taken on the metallic Au species. This effect may be related to the presence of very small Au NPs/clusters with less pronounced interference patterns (oscillations in the spectra) or by a small negative charge on Au. Evidence for Au^δ-^ species has been previously reported and is explained by the reduced support donating electron density to interfacial Au sites.^13−15^

Following this long deactivation measurement, we tested the catalyst after a subsequent calcination step (45 min in 10% O_2_/N_2_ at 200 °C). The reaction measurement after the calcination step (WGS-II) indicated a full conversion for around 10 min on-stream (see Figure S2). The essentially complete reversibility of the deactivation was surprising, given the evidence that the catalysts deactivate via particle growth. Spectra recorded during this reaction phase (WGS-II, first spectrum taken 1 min after the reaction was initiated, Figure S4) showed an almost similar oxidation state of Au as during reaction, suggesting that the bulk of the Au was not restored to its prereaction oxidation state. Hence, also in this phase metallic Au is likely to be the WGS-dominant active species. Interestingly, it was previously reported that a mixture of 5% O_2_/N_2_ at 150 °C did not restore catalytic activity or reoxidize the Au, whereas at 400 °C the Au was reoxidized.^16^

Therefore, we analyzed the EXAFS region to identify possible changes in the bonding environment around the Au species. EXAFS data of the fresh Au/CeZrO_4_ catalyst indicated a significant proportion of oxidized Au species with strong contribution from the Au–O backscattering shell appearing at 2.0 ± 0.02 Å, with an average Au–O coordination number of 2.80 ± 0.3 (see Figure S3, and Table S1). Also, we observed an additional contribution from the Au–Au shell (at 2.74 ± 0.02 Å and CN_Au–Au_ = 2.2), which can be attributed to a small fraction of Au clusters or Au–Au in oxidic particles.^17,18^ After heating the catalyst in N_2_ to 150 °C, no significant changes in the scattering shell or CN values for Au could be detected (see EXAFS parameters Table S1).

EXAFS data taken after 5 min (spectrum takes 1 h) from the start of the reaction showed a major contribution from the Au–Au shell (*R*_Au–Au_ = 2.72 ± 0.03 Å and CN_Au–Au_ = 3.5) with a very small shoulder from Au–O scattering at a Au–O distance of 1.97 ± 0.02 Å and very small coordination number (CN_Au–Au_ = 0.1 ± 0.1) (see Figure S4 and parameters in Table S1). With time on-stream, we observed an almost continuous increase of the average Au–Au contribution with the presence of a persisting, but very small contribution of Au–O (CN_AuO_ < 0.1) to the Fourier transformed data, reaching a CN_Au–Au_ of 5.8 after reaction for 590 min on stream (Figure S4b and Table S1). The observed increase of the first shell CN_Au–Au_ fits well with an observed increase in the Au–Au bond distance (from 2.72 Å in the first spectrum to 2.85 Å after 590 min), in good agreement with the expected increase of the bond length for bigger particles.^19^ The increase in the Au particle size on Au/CeZrO_4_ is consistent with our previous investigation into the deactivation of the Au/CeZrO_4_ under the same conditions.^6^

The analysis of the EXAFS data obtained on the catalyst during a second WGS step (WGS-II) directly after the calcination step at 200 °C indicated a significant decrease of the Au–Au coordination number from 5.9 at the end of first reaction phase (WGS-I) to 3.3 at the beginning of the second reaction phase (WGS-II), carried out after the oxidative treatment (see Figure S4c and Table S1). This result strongly indicates that under the oxidative treatment, the Au NPs were partially redispersed to form smaller particles, decrease in the bond length (see Table S1). Subsequent agglomeration of the Au during the second reaction phase (WGS-II) was then observed, with the CN_Au–Au_ reaching a value of 4.9 after 195 min on-stream. Such redispersion has not previously been observed for supported gold catalysts under oxidizing conditions; in fact, opposite findings had been published,^11^ and so we employed additional techniques to verify these observations.

Stop-start scanning transmission electron microscopy was previously used to visualize individual Au NPs after exposure to pretreatments and WGS reaction conditions.^6^ It was shown that subnanometer Au clusters and atomically dispersed species rapidly grew into Au NPs 1–2 nm in size after exposure to WGS conditions, forming the active species. Although these data provided insights into the activation and deactivation of Au/CeZrO_4_ during WGS conditions, *ex situ* STEM measurements involve imaging samples under ultrahigh vacuum. Since it has been shown that the shape of NPs is sensitive to the environment,^20^*in situ* transmission electron microscopy (*in situ* TEM) is better suited to identify changes in the catalyst structure during chemical reactions. We therefore employed this approach to observe changes to individual Au species *during* the WGS reaction. The experimental approach of the *in situ* TEM measurements is summarized in Figure S6. The reaction conditions similar to those in the fixed-bed flow reactor and the *operando* XAS experiments, but we also increased the reaction temperature to 250 °C to accelerate the aging of the catalyst. Of particular interest was the change in catalyst structure during the oxidative treatment after the WGS reaction.

**Figure 2:**
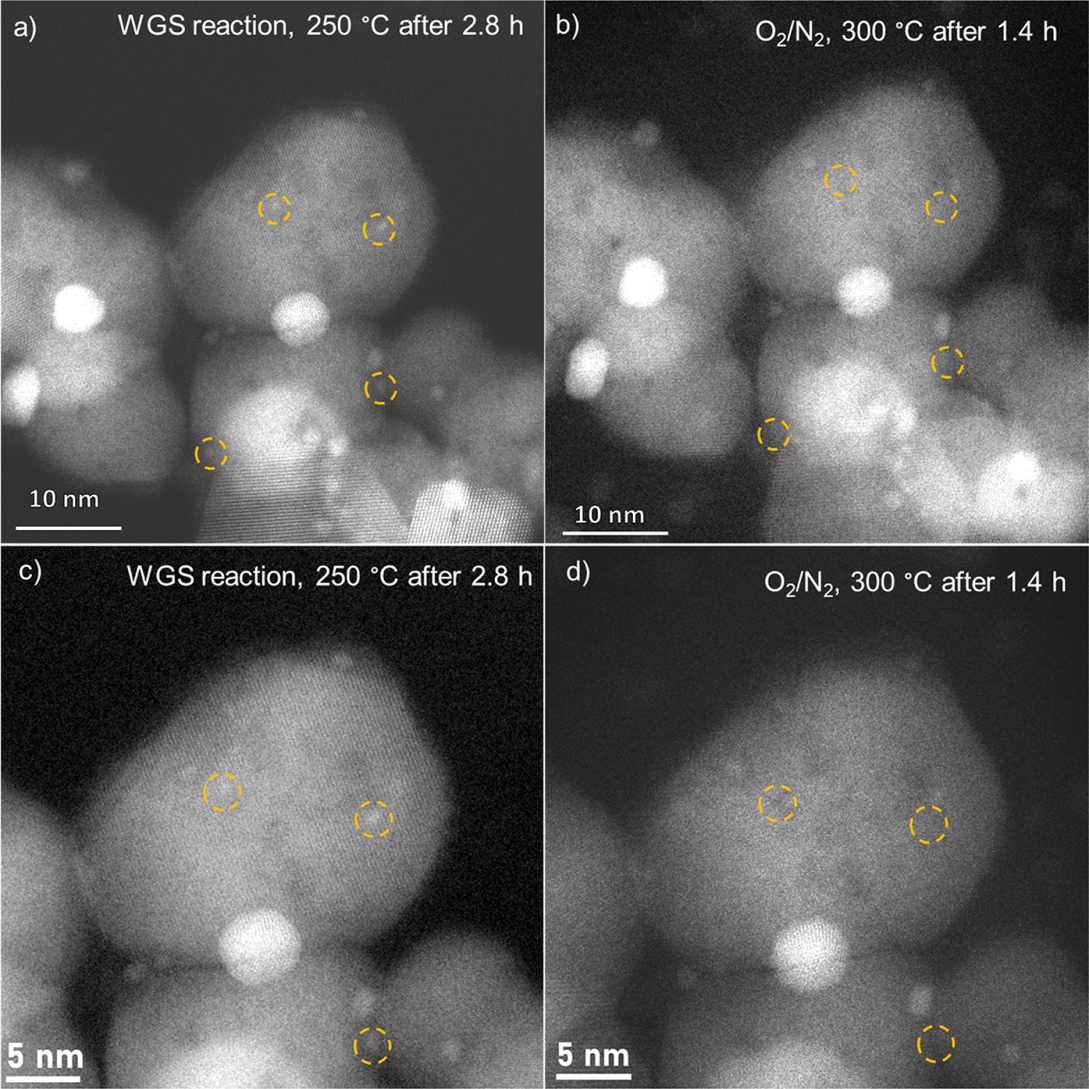
*In situ* HAADF STEM images of the 2.5 wt % Au/CeZrO_4_ during the WGS reaction (a and c) and during the oxidative regeneration step (b and d). The orange circles indicate particles that were present during the WGS reaction and then disappeared during the O_2_ treatment.

*In situ* HAADF STEM imaging of the Au/CeZrO_4_ catalyst before reaction revealed that this contains Au NPs with diameters in the range of about 0.5 to 10 nm, consistent with our previous findings.^6^Figure S7a–d shows various locations that were imaged under vacuum. Due to the poor mass contrast between Au and the CeZrO_4_ support, it is challenging to clearly identify particles below approximately 0.5 nm in diameter and not possible in this experiment. In the current work, we focused on the change in Au NPs during the change from WGS reaction conditions to oxidative conditions, where the *in situ* XAS measurements indicated that Au redispersion may occur. Figure 2a–d shows representative *in situ* HAADF STEM images of the Au/CeZrO_4_ catalyst taken during the WGS reaction at 250 °C (a and c) and during the oxidative regeneration step (b and d). Note that these images were taken from the same location, allowing direct comparison of changes in individual Au NPs. Figure 4c,d is taken from a higher magnification in order to better show the changes in Au NPs after oxidative treatment. When the reaction mixture was switched to 10% O_2_/N_2_, some of these particles disappeared after 1.4 h (see those in orange circles), providing further evidence that Au NPs can redisperse to smaller NPs (see particles circled in red).

**Figure 3:**
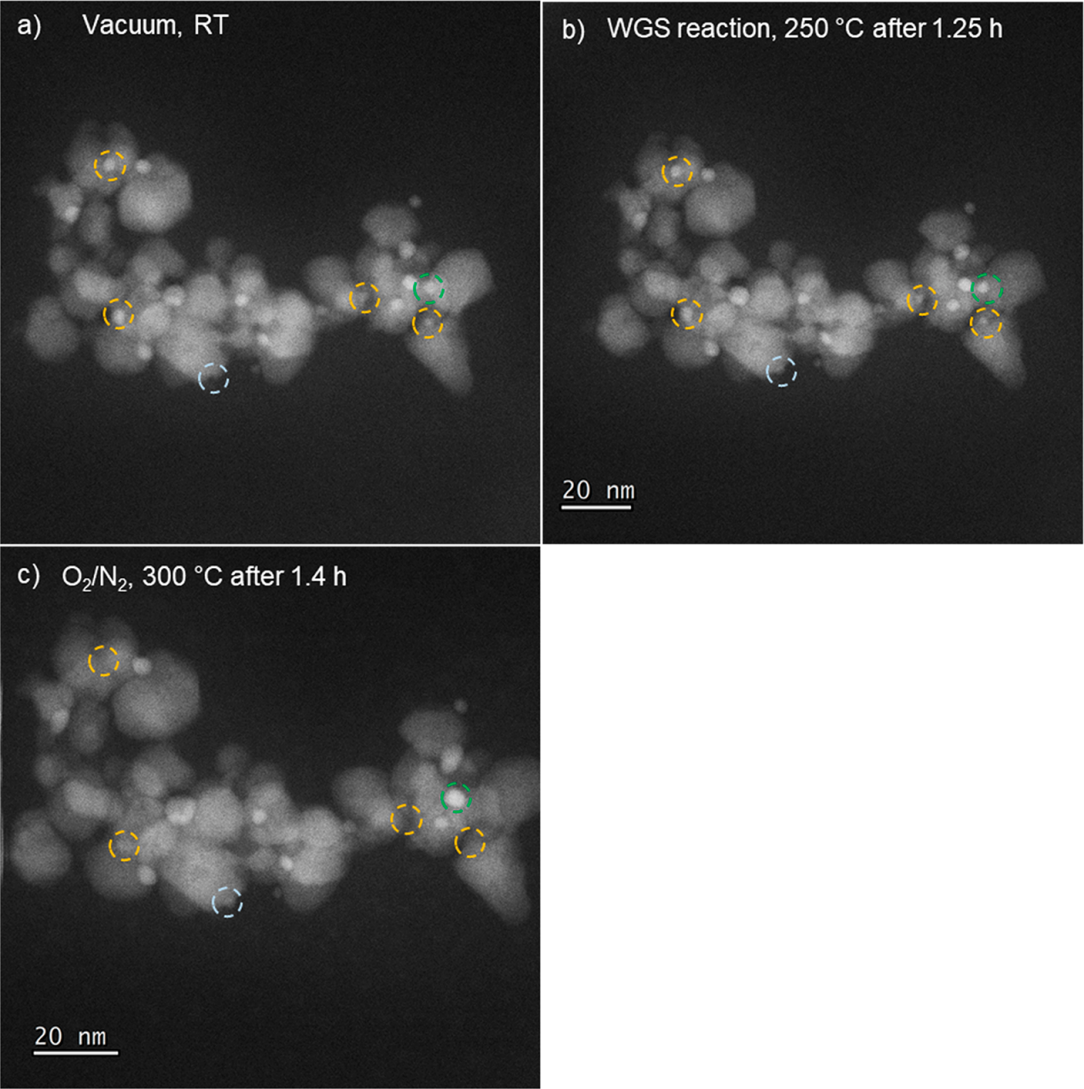
Additional *in situ* HAADF STEM images showing Au redispersion in the 2.5 wt % Au/CeZrO_4_ catalyst: (a) under vacuum at room temperature; (b) during the WGS reaction at 250 °C, and (c) during the oxidative regeneration treatment at 300 °C. The light blue circle shows a particle that was not present in the fresh sample, i.e., it formed during the heating of the catalyst bed or the early stages of the WGS reaction. The orange circles indicate particles that were present in the fresh catalyst and the green circle shows a particle that was stable under WGS conditions but then grew in size under the oxidative treatment.

Figure 3 shows an example of Au redispersion (see orange circles) alongside a nanoparticle that merged with another particle (see green circle) under the same conditions. Figures S8 and S9 provide additional examples of Au NPs that redisperse during the oxidative heat treatment (as well as an example of a particle that sinters (green circle, Figure S9). The particles that were seen to redisperse were below 2 nm in size, while all of the large NPs (>10 nm) and some of the smaller NPs, appeared unchanged after the oxidative regeneration, though it may not be possible at the current resolution to observe small changes to larger NPs. These findings clearly indicate that both redispersion and growth of Au can occur concurrently during the oxidative heat treatment. These local observations are in full agreement with the XAS results, confirming that the overall effect of the oxidative heat treatment was to redisperse the Au NPs, creating very small Au NPs in addition to further growth of larger NPs.

Observations of Au redispersion have been made before: Hernández et al. reported that for Au/CeEuO_X_ (but not Au/CeO_2_) activation in H_2_ led to an increase in the Au dispersion. This was concluded from the disappearance of Au reflections in X-ray diffraction and an increase in activity in CO oxidation.^21^ These authors suggested that O vacancies formed in the CeEuO_X_ support during the reductive heat treatment provide nucleation sites for Au. Romero-Sarria et al. previously reported that under a CO atmosphere, Au nanoparticles on high-surface-area CeO_2_ redispersed in a similar manner as the Au/CeEuO_*x*_ catalyst.^22^ Following earlier work,^23^ they also suggested that the O vacancies formed served as nucleation sites for Au. Due to the extended time required to obtain the XAS spectra (around 1 h), these data alone could not precisely identify when exactly the redispersion of the Au occurred, during the recalcination or in the initial phase of the WGS reaction (WGS-II). However, our *in situ* STEM results clearly show that redispersion of the Au NPs occurs during the oxidative treatment. Oxidative redispersion of noble metals such as Pd, Pt or Ru has been reported, but this effect is understood to be contingent on the stability of the metal oxide.^24,25^ Among different mechanisms, we tentatively propose that the interaction of oxygen with undercoordinated Au sites of the few nanometer-sized Au particles (as observed in the ETEM measurements) results in the weakening of Au–Au bonds of these sites. Subsequently thermally active oxidized periphery sites of Au can be detached and migrate on the surface under a flow of oxygen at 300 °C to reform the small Au NPs. Although there was no direct indication from our *operando* XAS measurements that Au oxidized during this oxidative step, the transient formation and decomposition of oxidized Au species cannot be excluded from these measurements.

**Figure 4:**
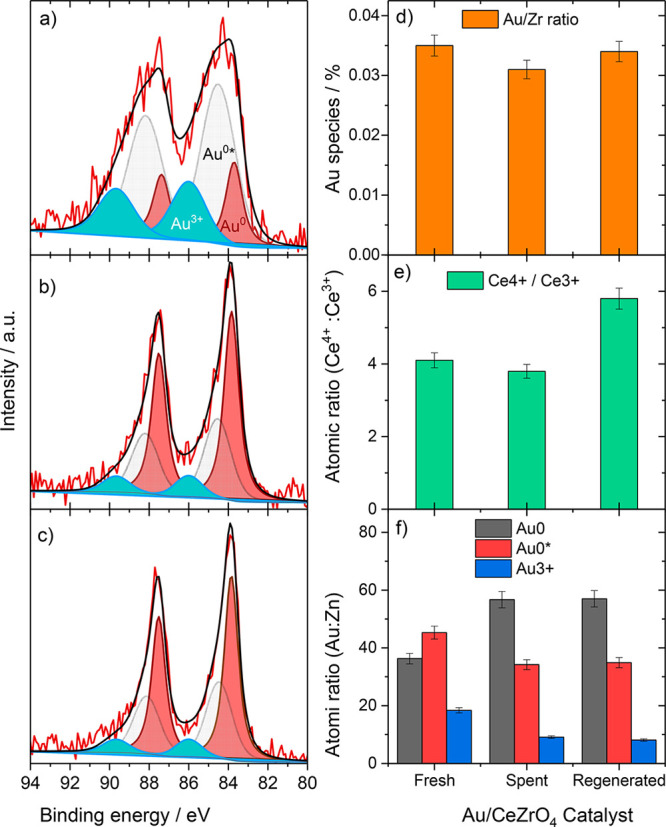
Au 4f region of (a) the fresh, (b) used, and (c) reoxidized (top) Au/CeZrO_4_ catalysts. Each sample contains Au^0^ (pink), Au^0^* (green), and Au^3+^ (light blue). (d) The Au/Zr ratio, (e) the Ce^4+^/Ce^3+^ ratio, and (f) the Au species (from quantification in a–c).

In order to further investigate the agglomeration and subsequent redispersion of Au, we carried out X-ray photoelectron spectroscopy (XPS) analysis of a fresh, used (for 6 h under the WGS reaction conditions described in the experimental section) and reactivated Au/CeZrO_4_ catalyst. Initially, the dispersion of Au was inferred by considering the atomic ratio of Au/Zr in the fresh, used, and regenerated catalyst, which is shown in Table 1 and Figure 4d (3p Zr region used for quantification is shown in Figure S10). A decrease in this ratio indicates an overall decrease in Au dispersion and an increase in this ratio indicates the opposite. There is a small decrease in the Au/Zr ratio from the fresh to the used sample, and a modest increase after regeneration. While it should be noted that the differences are small (about 10% of relative values), this trend is consistent with the XAS and *in situ* STEM data and supports the hypothesis that Au initially agglomerates under WGS conditions and then a fraction redisperses during the oxidative regeneration step.

**Table 1:** Quantification of the Au 4f Region of Each Sample and the Atomic Ratio of Au/Ce

sample identifier	Au/Zr ratio	species	binding energy (eV)	Au species atom %	Ce^4+^/Ce^3+^
**Au CeZrO**_**4**_**Fresh**	0.035	Au^0^	83.7	36.3	4.1
		Au^0^*	84.5	45.3	
		Au^3+^	86.0	18.4	
**Au CeZrO**_**4**_Used for 6 h	0.031	Au^0^	83.8	56.7	3.8
		Au^0^*	84.5	34.2	
		Au^3+^	86.0	9.1	
**Au CeZrO**_**4**_**Oxidized**	0.034	Au^0^	83.9	57.0	5.8
		Au^0^*	84.5	34.9	
		Au^3+^	86.0	8.1	

To examine changes in the Au speciation in more detail, the Au 4f region was analyzed and deconvoluted, once for fresh, used and regenerated samples (Figure 4a–c,f) In all three cases, the spectra were fit with peaks at about 83.9, about 84.5, and at 85.8 eV. It should be noted that differential charging led to a slight broadening of the peaks, in particular for the fresh catalyst. Such broadening, which can be a function of many factors including support particle size, means caution should be exercised in data interpretation. The first peak has previously been assigned to Au^0^ species.^6,26^ A peak at about 85.5 eV has been ascribed to small Au nanoparticles (<1 nm)^11,27−31^ and denoted Au^0^*, as well as to Au^+^ species.^32,33^ Given the abundance of small nanoparticles in the Au/CeZrO_4_ catalyst, this peak was labeled as Au^0^*. The third peak is characteristic for Au^3+^. It is typically observed in fresh Au/CeZrO_4_,^6,26^ but present in small amounts also during reaction and after reoxidation. The spectrum of the fresh catalyst is dominated by the Au^0*^ peaks, and a rather high contribution of Au^3+^ species, in good agreement with expectations for a catalyst obtained directly after drying, and also with the XANES results for a fresh catalyst. The significantly larger width of the Au^3+^ and Au^0*^ peaks in this catalyst we ascribe to an inhomogeneous composition, i.e., to the presence of slightly different species, which results in small differences in the binding energy and thus further broadens these peaks, in addition to differential charging effects. In the used sample, the proportions of Au^0^* and also of Au^3+^ decrease significantly, with Au^0^ dominating the spectrum, in good agreement with observations for Au/CeO_2_.^11^ This is consistent with further particle reduction and particle growth under reaction conditions. After the oxidative treatment the sample shows a comparable abundance of Au^0^* and also of Au^3+^ species as during reaction. This fully agrees with our XANES results, which also did not resolve an increase in oxidic Au species upon recalcination. Typically, Au^3+^ is observed in the as-prepared catalyst and is present due to incomplete reduction of the Au precursor, Au(OH)_3_. Under reaction conditions and during the heating of the catalyst bed, this reduces into small Au nanoparticles, which are highly active for the WGS reaction.^6,10^ Therefore, the role of the oxidative regeneration step may be to form small, oxidized Au species from Au nanoparticles, which would likely diffuse across the surface and anchor elsewhere on the support, e.g., on oxygen vacancies, which are known to stabilize Au nanoparticles.^34^ During the early stages of the second WGS reaction (or during the heating of the regenerated catalyst), these Au^3+^ species could form small, highly active Au nanoparticles. Consideration of the Ce^3+^/Ce^4+^ ratio shows that the support is slightly reduced during the WGS reaction and then reoxidizes after the oxidative regeneration step (Figures 4e and S11).

## Conclusions

In conclusion, we have demonstrated that it is possible to regenerate Au/CeZrO_4_ catalysts for the low-temperature WGS reaction via an oxidative heat treatment that redisperses the Au NPs. *Operando* XAS and *in situ* HAADF STEM were used to follow the dynamic changes of the nanoparticles during the reaction and after regeneration. Combining these techniques with activity measurements, we have confirmed that the highly active species, small Au^0^ NPs, are formed in the early stages of the WGS reaction (first 10 min) and that these grow subsequently during time on-stream. In addition to small Au NPs that are present in the early reaction, we consider the presence of clusters (0.5–1 nm) to also be catalytically relevant and, like CO oxidation, a hierarchy of activity may be present. The Au NPs can be partially redispersed by an oxidative regeneration step that forms very small, highly active Au NPs/clusters and restores the catalytic activity The direct evidence of Au redispersion also revealed the complexity of Au NP dynamics under reaction conditions. This understanding is a significant step toward developing robust catalysts that are capable of operating under extended periods.

## Methods

### Catalyst Preparation

The 2.5 wt % Au/CeZrO_4_ catalyst was prepared by a deposition-precipitation method. In a typical preparation, HAuCl_4_ (2.04 mL of a 12.25 mg mL^–1^ aqueous solution) was added to deionized water (200 mL). The CeZrO_4_ support (0.975 g, 1:1 Ce/Zr ratio, Solvay), was added to this solution and allowed to equilibrate for 15 min. Then, Na_2_CO_3_ (0.05 M) was added dropwise until the pH reached 8. The mixture was stirred vigorously for 1 h before being filtered under vacuum and washed with deionized water (800 mL). The recovered catalyst was dried for 6 h at 110 °C in static air.

### Kinetic Measurements

LT-WGS activity measurements were performed in a fixed bed quartz tube flow-reactor (inner diameter 4 mm and length 21 cm) at a temperature of 150 °C at atmospheric pressure, using high purity gases (99.999%) supplied by Westfalen AG. A semirealistic water gas mixture (2% CO, 8.1% H_2_, 7.5% H_2_O and balance N_2_) was prepared using Hastings HFC-202 mass flow controllers. Water vapor was introduced into the feed gas by bubbling the dry gas mixture (2% CO, 8.1% H_2_, and balance N_2_) at a total flow rate of 50 N mL min^–1^ through a bath of distilled water at constant temperature. The pure Au/CeZrO_4_ catalyst (58 mg) was loaded into the center of the microreactor and delimited from both sides by two pieces of thermally stable and catalytically inactive quartz wool. Influent and effluent gases were analyzed by online gas chromatography with a CO detection limit of <10 ppm (DANI 86.10), using H_2_ as carrier gas. The Au mass-normalized reaction rate was calculated from the CO conversion (X_CO_) under differential reaction conditions, using the molar flow rate of CO into the reactor (*ṅ̇*_CO,in_), and the absolute mass of Au metal in the catalyst (m_Au_) according to eq 1. Since the CO conversion was at the upper end of differential reaction conditions, between 29 and 19%, the actual rates may differ slightly, which, however, does not affect the conclusion of this work. To quantify and examine the deactivation with time on stream the relative (*R*_rel_) rate was calculated as a ratio of rate at different times (*R*_t_) and initial rate (*R*_in_, first data point collected after 5 min of reaction) as indicated in eq 2
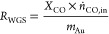
1
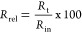
2

### *Operando* X-ray Absorption Spectroscopy (XAS)

Measurements were carried out at the Au L_III_ edge (11919 eV) at the XAFS beamline (storage ring Elettra-Trieste; Italy), making use of a Si (111) double crystal monochromator. For the *operando* XAS measurements a specially designed fluorescence reaction cell was used, which is described in more detail elsewhere.^10^ The measurements were carried out using 60 mg of pure nondiluted Au/CeZrO_4_ and a similar reaction gas mixture as described above. Together with the XAS measurements, the conversion was followed by analyzing the gas phase signals of CO using infrared spectrometry in a home-built analysis system consisting of a Bruker Alpha single beam transmission IR-spectrometer and a substrate integrated hollow waveguide (iHWG).^35^ IR spectrometry measurements allowed for higher time resolution (1 min per run) during WGS measurements, which helped us to monitor the initial activation phase. Spectra of reference materials of a Au foil and Au_2_O_3_ were collected at the Au L_III_ edge for data evaluation. Background subtraction, normalization and energy shifts analysis of the XANES spectra were performed using the Athena software (IFEFFIT program package).^36^ Data reduction and fitting of the EXAFS spectra were carried out using the XDAP software package with standard procedures described in detail elsewhere.^37,38^ Theoretical references were calculated by the FEFF 8.0 code and calibrated using spectra of Au foil and Au_2_O_3_ experimental references.^37,39^ EXAFS data were evaluated in *R*-space using fixed *k* and *R* ranges (*k*, 2.6–8.5 Å^–1^; *R*, 0–4.5 Å). Data evaluation was first done using multiple *k*-weightings (*k*^1^, *k*^2^ and *k*^3^) to avoid artifact in the evaluation of parameters due to error in background subtraction or normalization (see Figure S5 and Table S2, SI).^40^ The data were fitted to the shortest backscatterer distance to Au (i.e., Au–Au and Au–O); the bond length, coordination number (CN), and internal energy shift (*E*_0_) were allowed to change freely. The Debye–Waller factor (DWF) was allowed to change. All XANES/EXAFS measurements during reaction were performed in the same reformate gas described as in section above.

### Closed Gas Cell *In Situ* Scanning Transmission Electron Microscopy

*In situ* STEM measurements under atmospheric pressure were performed on a Thermo-Fisher Themis Z double-corrected S/TEM, which operated under STEM mode at 300 keV and was fitted with a DENSsolutions Climate gas holder. In order to recreate the conditions of a typical WGS reaction, the sample was exposed to several different environments. First, the sample was imaged under vacuum at 150 °C. Then, the WGS mixture (2.5 vol % H_2_O, 2.7 vol % H_2_, 0.67 vol % CO, 0.67 vol % CO_2_ + N_2_ to balance) was introduced to the sample at the same temperature. Afterward, the temperature was gradually increased to 250 °C for about 3 h. After the reaction mixture was switched to O_2_, the sample was treated at 250 °C for about 90 min. Finally, the temperature was raised to 300 °C while maintaining the same gas mixture for 90 min. The total pressure in the sample chamber was 800 mbar. During the whole experiment, we opened the column valve occasionally to image different areas. The sample was under the dark environment during the rest of experimental times. No beam effects, including beam-induced redispersion or aggregation, were observed during the *in situ* STEM experiment.

### X-ray Photoelectron Spectroscopy (XPS)

Spectra were recorded using a Kratos Axis Ultra-DLD photoelectron spectrometer utilizing monochromatic Al K_α_ radiation operating at 144 W (12 mA × 12 kV) power. High resolution and survey scans were performed at pass energies of 40 and 160 eV, respectively, with respective step-sizes of 0.1 and 1 eV. A magnetically confined low energy electron charge compensation system was used to minimize sample charging and the resulting spectra were calibrated to the lowest C(1s) line, taken to be 284.8 eV. Spectra were fitted using a Voigt-like function using CasaXPS v2.3.24.^41^

## Supporting Information Available

Additional reaction data, EXAFS measurements and fitting parameters, *in**situ* TEM parameters, additional *in**situ* TEM images and additional XPS figures (PDF)
