# Supplementary material for: Reversible Growth of Gold Nanoparticles in the Low-Temperature Water–Gas Shift Reaction
Source: ACS Nano. 2022 Aug 25;16(9):15197–205. doi: 10.1021/acsnano.2c06504 (PMC9527796; doi:10.1021/acsnano.2c06504)
Supplement: Supplementary file 1 — nn2c06504_si_001.pdf [file nn2c06504_si_001.pdf]

Supplementary Materials for

Reversible growth of gold nanoparticles in the low-temperature water-gas shift reaction

James H. Carter[1]*, Ali M. Abdel-Mageed[2,3]*✉, Dan Zhou[4], David J. Morgan[1], Xi Liu[5]✉, Joachim Bansmann[2], Shilong Chen[2,§], R. Jürgen Behm[2,†] and Graham J. Hutchings[1]✉

1. Max Planck-Cardiff Centre on the Fundamentals of Heterogeneous Catalysis FUNCAT, Cardiff Catalysis Institute, School of Chemistry, Cardiff University, Cardiff, CF10 3AT, UK.
2. Leibniz Institute for Catalysis (LIKAT Rostock), D-18059 Rostock, Germany.
3. Institute of Surface Chemistry and Catalysis, Ulm University, Albert-Einstein-Allee 47, D-89081, Ulm, Germany.
4. DENSsolutions B.V., Delft 2628 ZD, The Netherlands.
5. *School of Chemistry and Chemical Engineering, In situ Center for Physical Sciences, Shanghai Jiao Tong University, 800 Dongchuan Rd. Minhang District, Shanghai, China, 200240.*
**These authors contributed equally*
[§] *Pres. address: Institute of Inorganic Chemistry, Kiel University, Max-Eyth-Straße 2, D-24118 Kiel, Germany.*
[†] *Pres. Address: Institute of Theoretical Chemistry, Ulm University, Albert-Einstein-Allee 11, D-89081 Ulm, Germany.*

✉ Corresponding authors: Graham J. Hutchings, hutch@cardiff.ac.uk; Ali M. Abdel-Mageed, Ali.AbdelMageed@catalysis.de and Xi Liu, liuxi@sjtu.edu.cn.

This file includes:
Figs. S1 to S11
Table S1

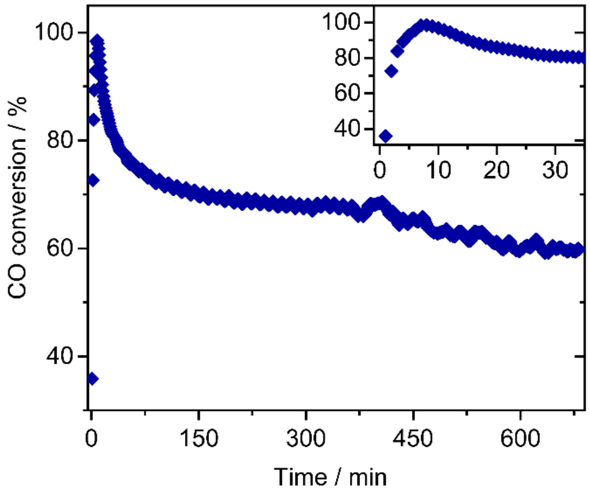

Figure S1. CO conversion during WGS reaction on the fresh-as prepared Au/$CeZrO_4$ catalyst at 150 °C in reformate gas (2% CO, 8.1% H_2, 7.5% H_2O and balance N_2 – 50 Nml min^{-1}) in the XAS reaction cell and using IR spectrometer (time resolution = 1 min). Inset: Initial activation and deactivation over 30 mins.

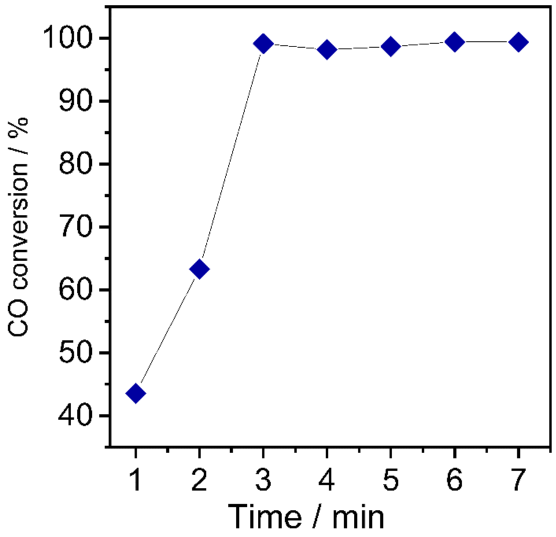

Figure S2. CO conversion during WGS reaction at 150 °C in reformate gas (2% CO, 8.1% H_2, 7.5% H_2O and balance N_2 – 50 Nml min^{-1}), after re-calcination of the spent Au/$CeZrO_4$ catalyst (measurement described in Fig. S1) in XAS reaction cell and using IR spectrometer (time resolution = 1 min).

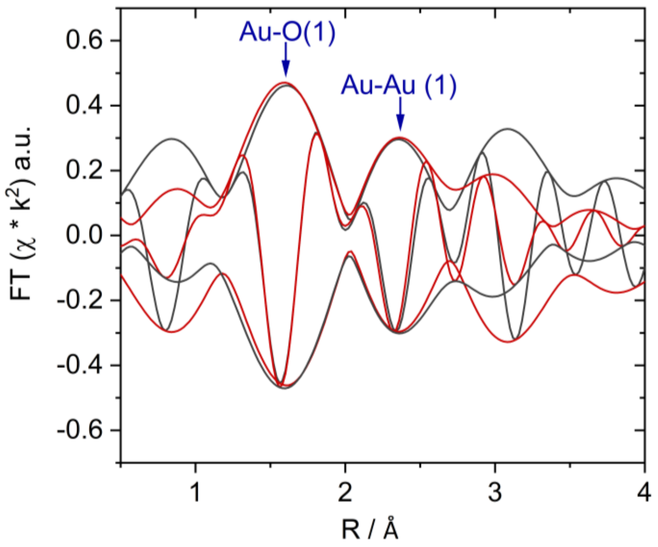

Figure S3. FT-EXAFS spectra (magnitude and imaginary part; black line: raw data; blue lines: fits) of the fresh as prepared Au/$CeZrO_4$ catalyst under flow of N_2 at 250°C. Data is phase shift-corrected. Comment: A contribution from the Au-Au shell (at 2.74 ± 0.02 Å and $CN_{Au\text{-}Au}$ = 2.2) for the fresh heated sample can be attributed either to a small fraction of Au clusters or Au-Au in oxidic particles [1,2].

Table S1. EXAFS data evaluation parameters of the native Au/$CeZrO_4$ and during WGS reaction at 150 °C.

Measurement	CN	s^2 / Å^2	R / Å	E_0 /eV
Fresh 25°C<br>(Au-O/Au-Au)	2.80 ± 0.30<br>2.2 ± 0.60	0.0096 ± 0.003<br>0.0130 ± 0.005	2.00 ± 0.014<br>2.74 ± 0.015	-7.7 ± 0.5<br>17.0 ± 5.0
Fresh @ 150°C<br>(Au-O/Au-Au)	1.9 ± 0.3<br>1.9 ± 1.8	0.009 ± 0.003<br>0.006 ± 0.007	1.99 ± 0.015<br>2.73 ± 0.018	-2.2 ± 0.5<br>16.0 ± 2.5
WGS-I - 5 min<br>(Au-O/Au-Au)	0.06 ± 0.06<br>3.5 ± 0.65	-0.007 ± 0.0035<br>0.010 ± 0.003	1.97 ± 0.015<br>2.72 ± 0.020	-8.9 ± 8.3<br>6.0 ± 1.0
WGS-I - 95 min	0.1 ± 0.1<br>3.4 ± 1.2	-0.010 ± 0.005<br>0.010 ± 0.0035	1.97 ± 0.015<br>2.72 ± 0.030	-3.8 ± 20<br>9.1 ± 4.8
WGS-I - 195 min<br>(Au-O/Au-Au)	0.06 ± 0.06<br>3.9 ± 1.1	-0.002 ± 0.005<br>0.0144 ± 0.004	1.97 ± 0.02<br>2.74 ± 0.030	-7.3 ± 20<br>6.19 ± 5.4
WGS-I - 320 min<br>(Au-O/Au-Au)	0.1 ± 0.1<br>4.4 ± 0.8	-0.02 ± 0.007<br>0.012 ± 0.006	2.0 ± 0.02<br>2.76 ± 0.022	-7.3 ± 20<br>6.19 ± 5.4
WGS-I - 410min	0.02 ± 0.05<br>5.0 ± 0.78	-0.03 ± 0.02<br>0.012 ± 0.006	1.96 ± 0.02<br>2.85 ± 0.02	-9 ± 5<br>-2.6 ± 3.0
WGS-I - 500 min<br>(Au-O/Au-Au)	0.02 ± 0.05<br>5.4 ± 0.70	-0.018 ± 0.006<br>0.012 ± 0.005	1.98 ± 0.022<br>2.85 ± 0.033	8.8 ± 19.8<br>-2.7 ± 1.8
WGS-I - 590 min<br>(Au-Au)	5.8 ± 0.9	0.014 ± 0.005	2.85 ± 0.022	8.0 ± 5.0
**After recalcination step in 10% O_2/N_2 at 200°C for 45 min**				
WGS-II - 5 min<br>(Au-O/Au-Au)	0.1 ± 0.1<br>3.4 ± 0.6	-0.05 ± 0.003<br>0.009 ± 0.003	1.97± 0.021<br>2.77 ± 0.025	10 ± 3.0<br>1.9 ± 1.1
WGS-II - 95 min<br>(Au-O/Au-Au)	0.03 ± 0.01<br>4.2 ± 1.2	-0.05 ± 0.007<br>0.015 ± 0.008	2.0 ± 0.03<br>2.77 ± 0.03	-6.3 ± 6.0<br>1.9 ± 1.1
WGS-II - 195 min<br>(Au-O/Au-Au)	0.03 ± 0.02<br>4.9 ± 1.0	-0.005 ± 0.005<br>0.015± 0.009	1.97 ± 0.022<br>2.82 ± 0.035	-5.5 ± 10<br>2.3 ± 3.7

[a]CN: Au−Au, Au−O first shell coordination number. [b] s^2 mean square displacement, part of the Debye-Waller factor (DWF: $\exp(-2s^2k^2$; k is wave vector). [c]R: Au−Au / Au-O first shell bond distance. [d]E_0: energy reference parameter.

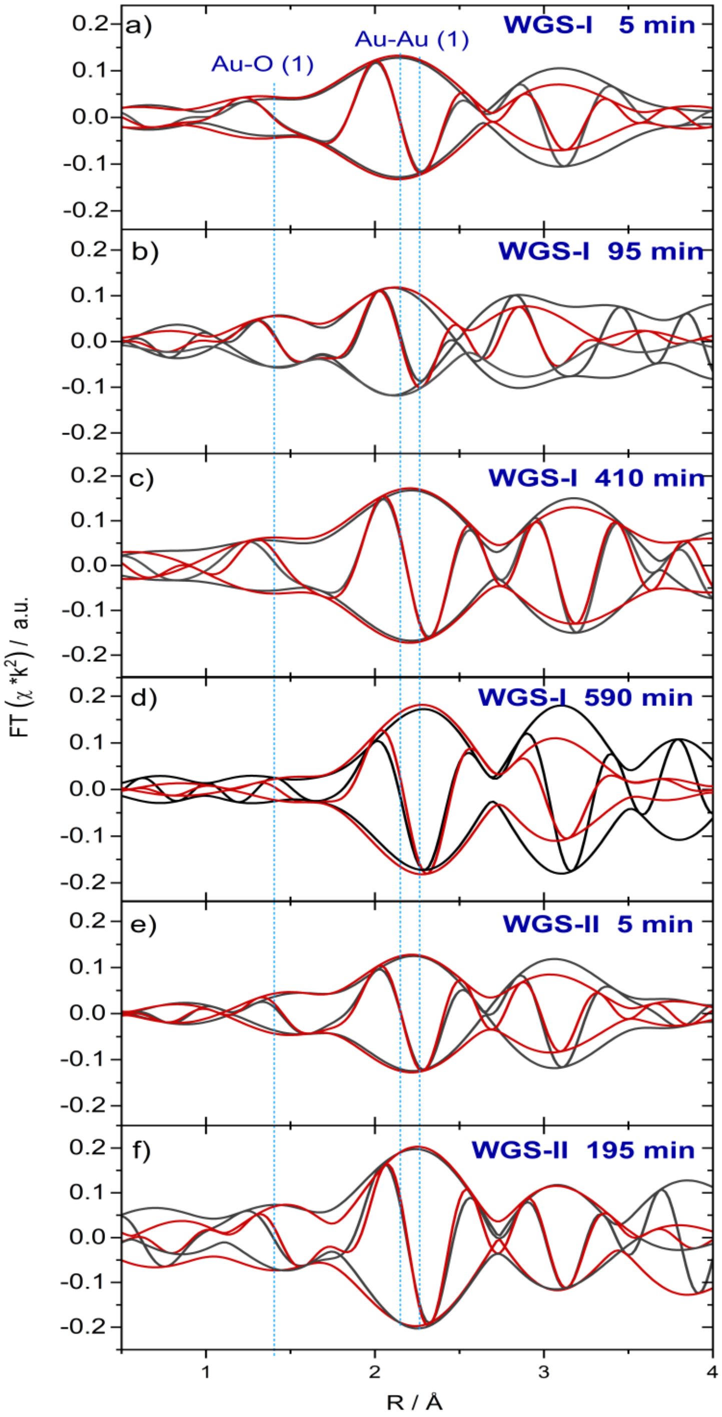

Figure S4: FT-EXAFS spectra (magnitude and imaginary part; black line: raw data; blue lines: fits) of Au/$CeZrO_4$ catalyst a) during WGS-I at 150°C (a: 5 min; b 95 min; c: 410 min; d: 590 min) and during the second WGS-II, after the oxidative treatment (e: 5 min; f: 195 min). Note that the duration of one single spectrum is 1 h, after the end of recording XANES region (6 min).

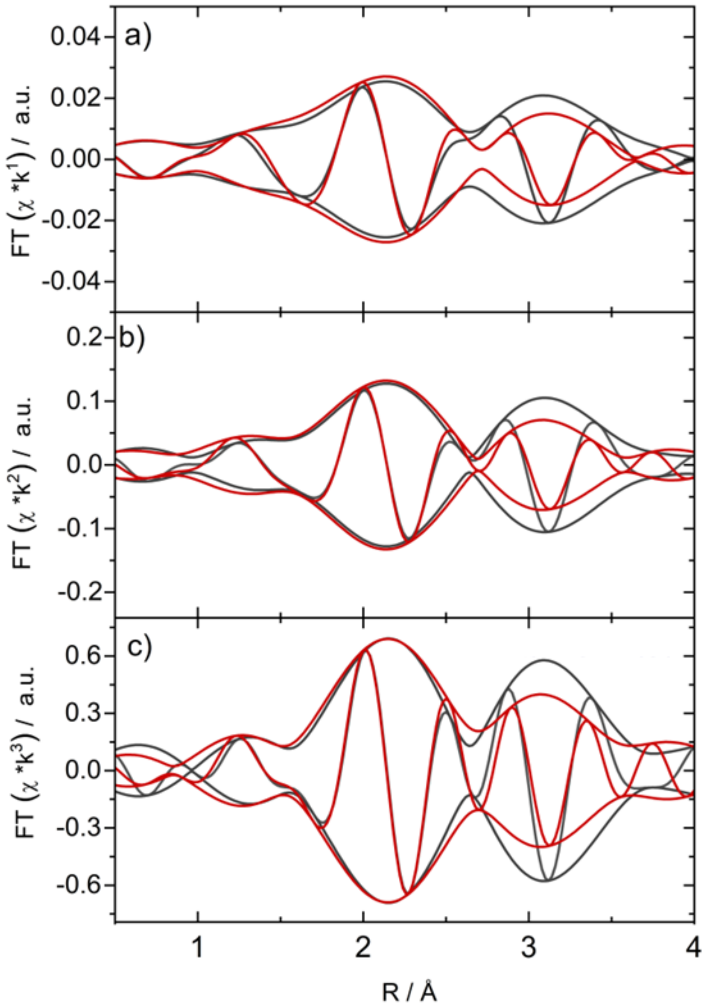

Figure S5. Comparison of a) k^1, b) k^2 and c) k^3 weighted FT-EXAFS data collected during the first WGS reaction at 150°C.

Table S2. Fit parameters of K1,K2 and K3 weighted FT-EXAFS data collected during the first WGS reaction at 150°C.

Measurement	CN	σ^2 / Å^2	R / Å	E_0 /eV
k^1-weighted	0.07 ± 0.22<br>3.3 ± 1.8	-0.0185 ± 0.004<br>0.0065 ± 0.005	2.09 ± 0.02<br>2.8 ± 0.0064	-8.9 ± 8.3<br>6.0 ± 1.0
K^2-weighted	0.06 ± 0.06<br>3.0 ± 0.65	-0.007 ± 0.0035<br>0.010 ± 0.003	1.97 ± 0.015<br>2.72 ± 0.020	-8.9 ± 8.3<br>6.0 ± 1.0
k^3-weighted	0.08 ± 0.06<br>3.2 ± 0.7	-0.007 ± 0.005<br>0.011 ± 0.005	1.966± 0.02<br>2.73 ± 0.015	-10.3 ± 15.6<br>0.64 ± 1.4

[a]CN: Au−Au, Au−O first shell coordination number. [b] σ^2 mean square displacement, part of the Debye-Waller factor (DWF: exp(-2$\sigma^2 k^2$; k is wave vector). [c]R: Au−Au / Au-O first shell bond distance. [d]E_0: energy reference parameter.

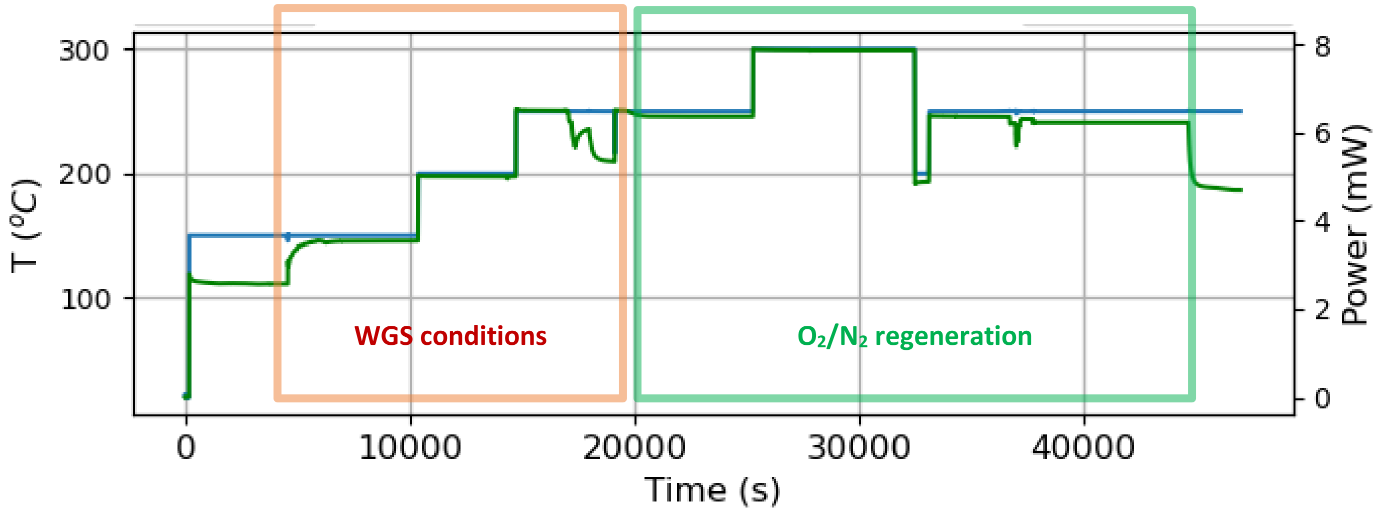

Figure S6. Summary of the time-line of the in situ STEM experiment.

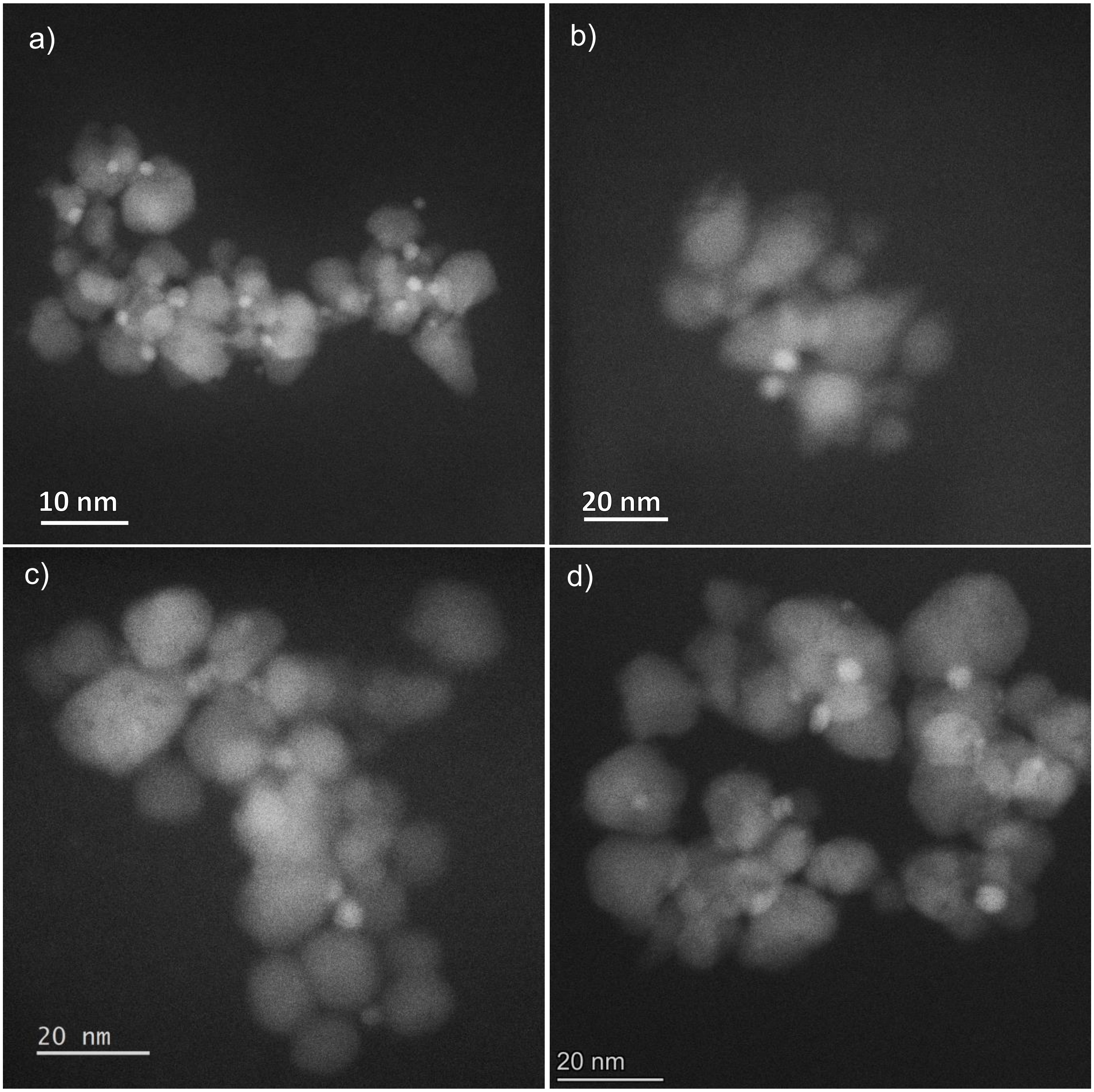

Figure S7. The Au/$CeZrO_4$ under vacuum at room temperature **(A-C)** and at reaction temperature, 150 °C, **(D)**. The fresh catalyst consists of a range of Au NPs from below 1 nm up to ca. 7-8 nm.

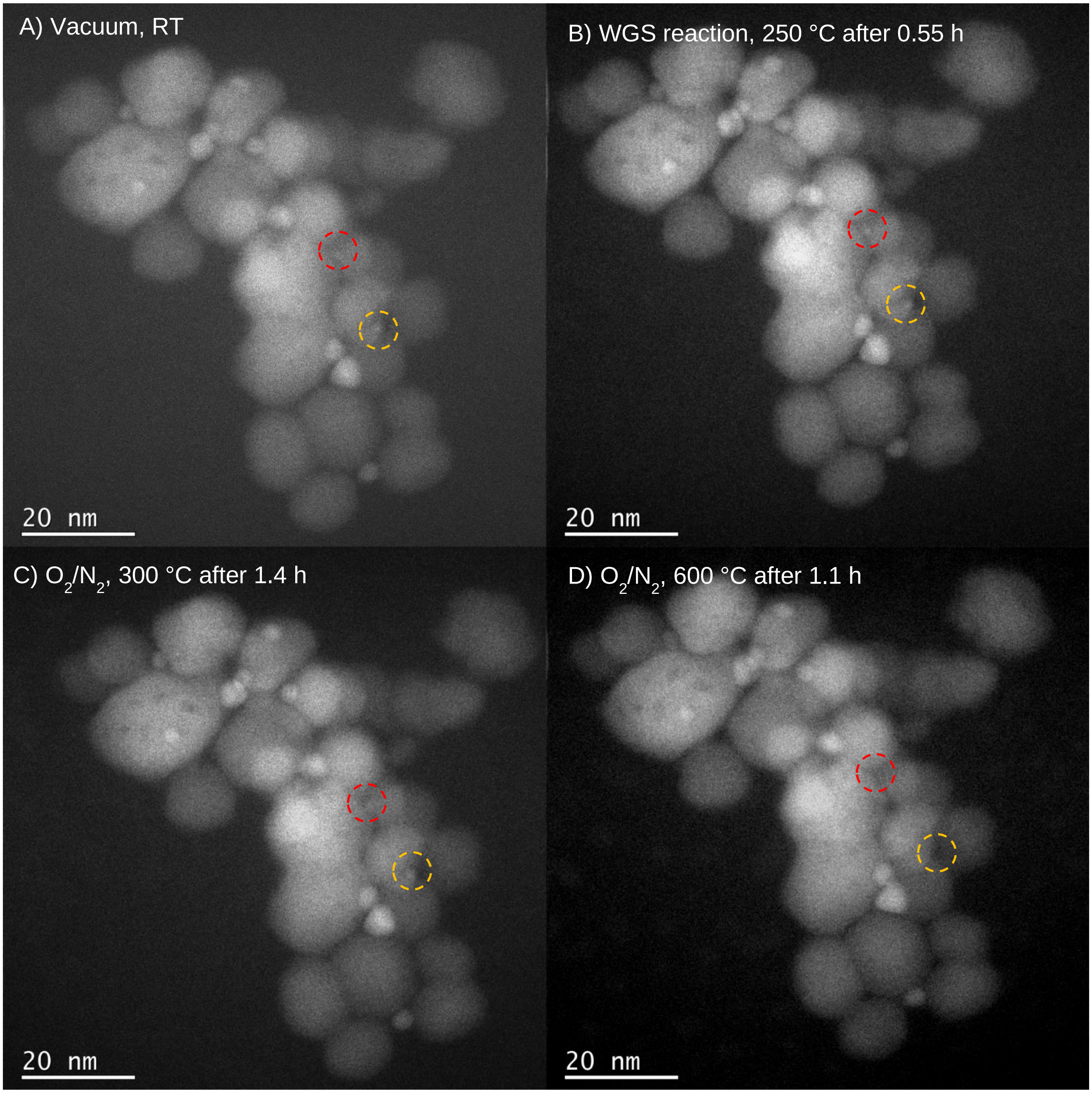

Figure S8. In-situ HAADF STEM images showing further evidence of Au re-dispersion in the 2.5wt% Au/$CeZrO_4$ catalyst: **A)** under vacuum at room temperature; **B)** during the WGS reaction at 250 °C, **C)** after 1.4 h in O_2/N_2 at 300 °C and **D)** after 1.1 h in O_2/N_2 600 °C. The red circles show a particle that was not present at RT under vacuum, and disappeared after 1.4 h under O_2/N_2 at 300 °C. The orange circles show a particle that was present in the fresh sample and disappeared after 1.1 h in O_2/N_2 at 600 °C.

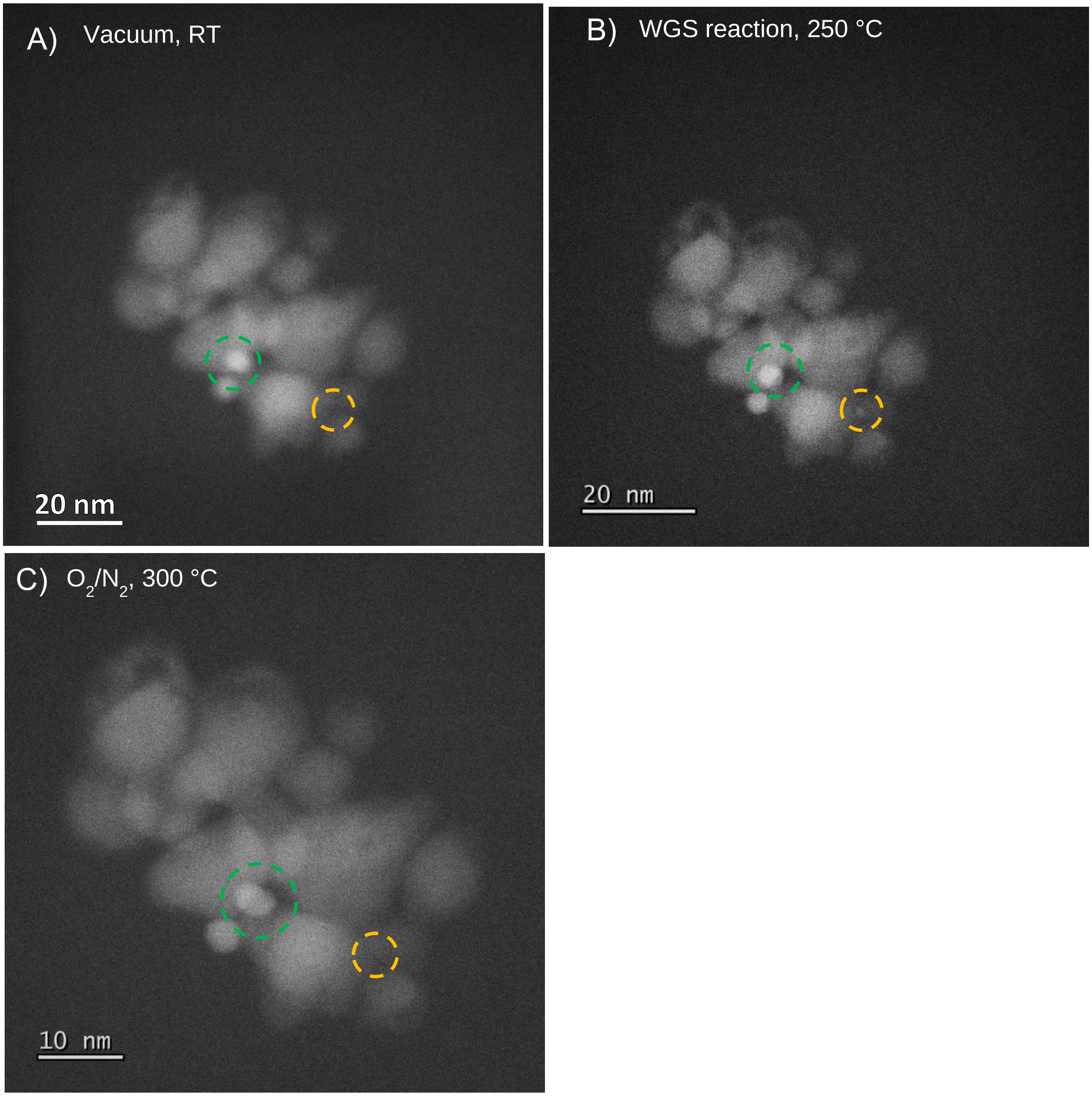

Figure S9. In situ HAADF STEM images of the 2wt% Au/$CeZrO_4$: **A)** Under vacuum at RT, **B)** during the WGS reaction at 250 °C and **C)** during the oxidative regeneration at 300 °C. The orange circle indicates an Au NP formed during the WGS reaction, while the yellow arrow indicates that the same NP has re-dispersed. The Au NP in the green circle indicates that some particle agglomeration also occurred under the oxidative regeneration step.

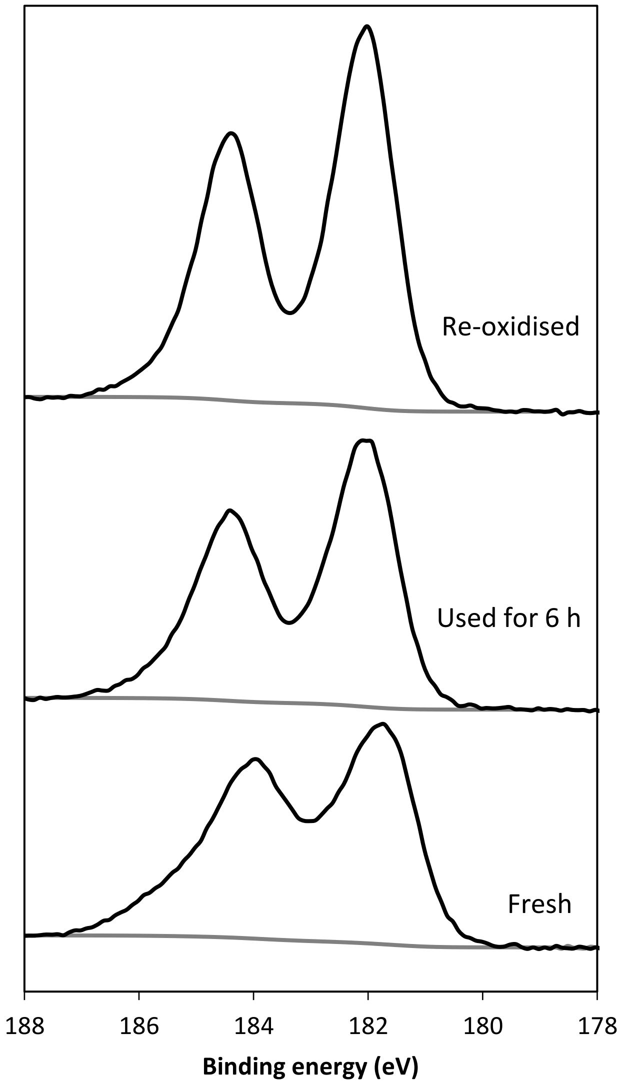

Figure S10. Zr 3d spectra for the samples quantified in Table 1.

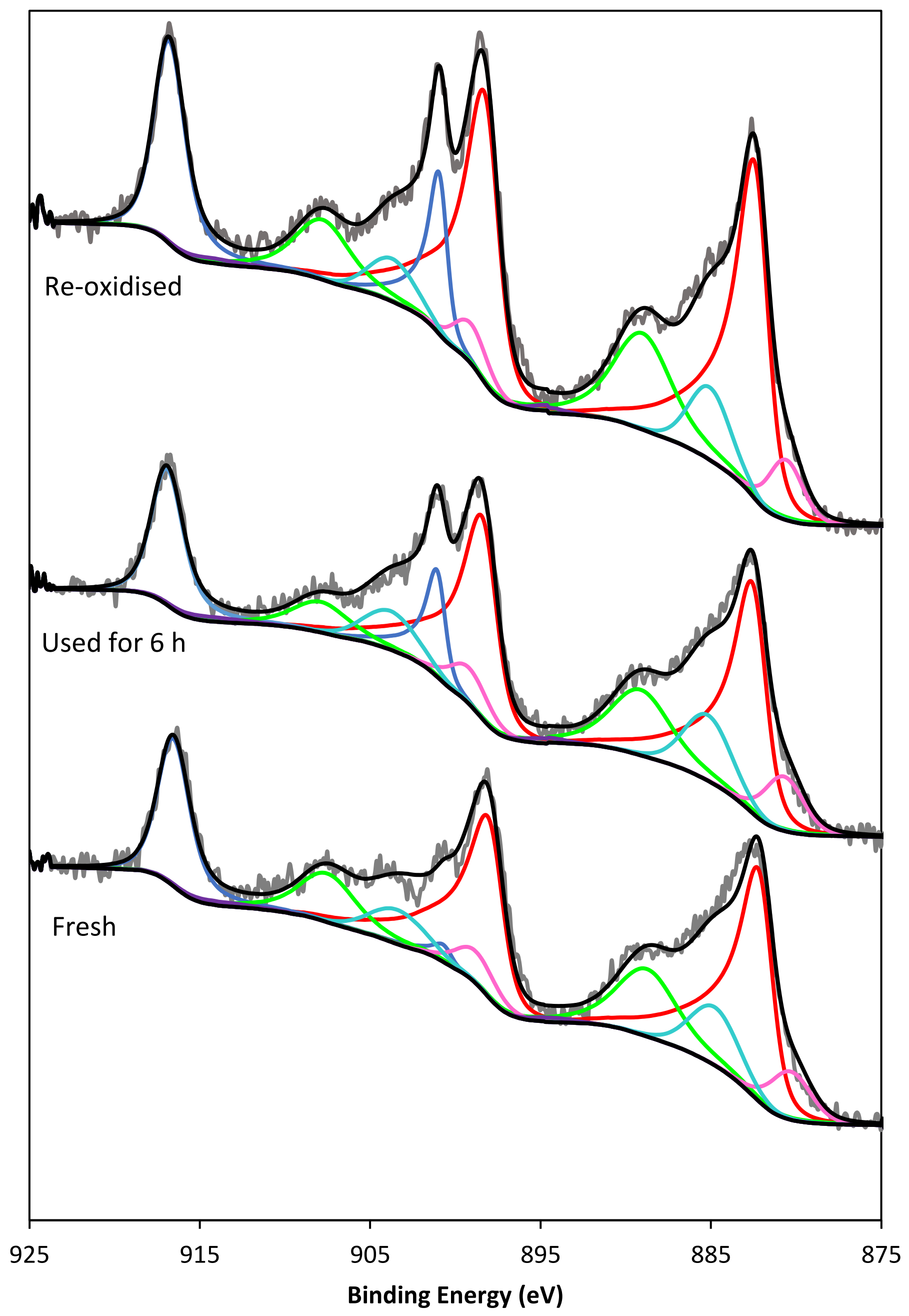

Figure S11. Deconvoluted Ce 3d spectra of fresh, used and re-oxidised $Au/CeZrO_4$ catalysts.

References

(1) Pantelouris, A.; Küper, G.; Hormes, J.; Feldmann, C.; Jansen, M. Anionic Gold in Cs3AuO and Rb3AuO Established by X-Ray Absorption Spectroscopy. *J. Am. Chem. Soc.* **2002**, *117* (47), 11749–11753.

(2) Weiher, N.; Bus, E.; Delannoy, L.; Louis, C.; Ramaker, D. E.; Miller, J. T.; Van Bokhoven, J. A. Structure and Oxidation State of Gold on Different Supports under Various CO Oxidation Conditions. *J. Catal.* **2006**, *240*, 100–107.
